# Pharmacological profiling of a dual FAK/IGF-1R kinase inhibitor TAE226 in cellular and in vivo tumor models

**DOI:** 10.1186/s13104-019-4389-7

**Published:** 2019-06-18

**Authors:** Shigemi Fukami, Daisaku Tomioka, Yutaka Murakami, Toshiyuki Honda, Shinji Hatakeyama

**Affiliations:** 1 Novartis Institutes for BioMedical Research, Novartis Pharma K.K, Tsukuba, Ibaraki Japan; 2 Novartis Institutes for BioMedical Research, Inc., Cambridge, MA USA; 3 Novartis Institutes for BioMedical Research, Novartis Pharma AG, Basel, Switzerland

**Keywords:** Focal adhesion kinase, Insulin-like growth factor-1 receptor, Cell proliferation, Anti-tumor

## Abstract

**Objective:**

A dual inhibitor of focal adhesion kinase (FAK) and insulin-like growth factor 1 receptor (IGF-1R), TAE226, was evaluated in a panel of cancer cell lines, MIA PaCa-2 human pancreatic tumor and 4T1 murine breast tumor models. The profiling data were generated during the drug discovery research prior to the first publication of TAE226 appeared in 2007 (Liu et al. in Mol Cancer Ther 6:1357–1367, 2007; Shi et al. in Mol Carcinog 46(6):488–496, 2007; Halder et al. in Cancer Res 67(22):10976–10983, 2007).

**Results:**

In a panel of 37 cancer cell lines, TAE226 showed a mean GI_50_ value of 0.76 μmol/L. In the MIA PaCa-2 model, TAE226 inhibited phosphorylation of Y397-FAK and phosphorylation of S473-Akt as IGF-1R signaling in the cell culture in vitro and the tumor in mice. Oral administration of TAE226 induced tumor stasis at 30 mg/kg and tumor regression at 100 mg/kg in the subcutaneous tumor, and inhibited the orthotopic tumor growth in a dose-dependent manner. Similarly in the 4T1 model, TAE226 inhibited phosphorylation of Y397-FAK and S473-Akt in the cell culture in vitro and the tumor in mice. Oral administration of TAE226 inhibited the orthotopic tumor growth and metastasis to the lung in a dose-dependent manner. Thus, TAE226 represents a novel class of selective and small molecule kinase inhibitor with a potent in vivo activity.

**Electronic supplementary material:**

The online version of this article (10.1186/s13104-019-4389-7) contains supplementary material, which is available to authorized users.

## Introduction

Focal adhesion kinase (FAK) is a non-receptor cytoplasmic tyrosine kinase that regulates multiple cell functions [1]. A number of evidences suggest that FAK plays important roles in cancer cell proliferation and survival [2, 3]. Elevated mRNA and protein expression levels of FAK have been reported in breast, colon, prostate, ovarian, invasive thyroid tumors, and esophageal squamous cell carcinoma correlating with invasive potential [4–11]. On the other hand, overexpressing a kinase-dead FAK mutant (FRNK) showed prevention of experimental tumor metastasis to lung of v-src transformed cells [12]. Moreover, silencing of FAK by small interfering RNA decreases tumor growth [13]. These data suggest that FAK is overexpressed in preinvasive lesions and is sustained in invasive and metastatic tumors where overexpression of FAK confers a selective advantage to survive apoptotic stimuli during metastasis.

Insulin-like growth factor 1 receptor (IGF-1R) is a trans-membrane tyrosine kinase receptor expressed in a wide variety of cell types. High levels of IGF-1R, and/or its activating ligands IGF-1 and IGF-2 have been associated with various types of human cancer (e.g. multiple myeloma, breast, prostate, colon, lung, pancreas, Ewing’s sarcoma) and also found to correlate with increased invasiveness and metastatic potential [14–16]. IGF-1R has been shown to be necessary for anchorage-independent growth and survival, as well as for transformation by several oncogenes. Consistently, interference with its function by inhibiting its expression, by preventing ligand binding with an antibody, or by blocking its signaling capacity with a truncated or kinase inactive form of the receptor has been shown to reduce tumor progression and/or metastasis formation in animal models [17, 18]. Furthermore, FAK is activated by IGF-1-mediated association of integrin β1 with IGF-1R in multiple myeloma cells grown in culture [19]. FAK and IGF-1R provide inputs into both Akt and MAPK pathway. Inhibition of both kinases should have a greater effect on important downstream effectors than inhibition of either enzyme alone.

Therefore, a dual inhibitor of both kinases may selectively block tumor growth, migration and survival of FAK- and IGF-1R-expressing tumor cells compared to proliferating and migrating normal cells. TAE226, a potent dual inhibitor of FAK/IGF-1R, has been described in literatures since the first publication of TAE226 appeared in 2007 [20–22] and its kinase selectivity has been also reported elsewhere [23, 24]. Here we report pharmacological profiles of TAE226 generated during the course of drug discovery research conducted until 2004.

## Main text

### Methods

#### Cellular proliferation assays

Sulforhodamine B assay was used for adhesive cell lines according to the procedure of National Cancer Institute [25]. Cell lines were cultured in RPMI 1640 supplemented with 10% heat inactivated fetal bovine serum and antibiotic–antimycotic solution at 37 °C with 5% CO_2_. Dependent upon cell doubling time, between 5000 and 20,000 cells were inoculated into 96 well microtiter plates in a volume of 100 µL per well. Plating density of each cell lines used in this study was listed in Additional file 1: Table S1. The plates were incubated at 37 °C, 5% CO_2_ for 24 h prior to the addition of TAE226. TAE226 was dissolved in DMSO at a concentration of 10 mM and stored in aliquots at − 20 °C. Prior to use, an aliquot of frozen concentrate was thawed and additional half-log dilutions (1:3.16) for a total of eight drug concentrations were prepared in DMSO. At time of drug addition, each dilution was further diluted to twice the desired final maximum test concentration with complete medium. After 24 h incubation, an aliquot of 100 μL of each drug dilution was added to the appropriate well in triplicate that already contains 100 µL of medium containing the cells. Time zero (Tz) control wells were fixed in situ by the gentle addition of 50 μL of 50% TCA to establish the cell population at time of drug addition.

The plates were then incubated for 48 h at 37 °C, 5% CO_2_. Cells were fixed in situ with 50% TCA and incubated for 60 min at 4 °C. Plates were washed three times with distilled water after the supernatant was discarded and air dried. Then, 100 µL of 0.4% Sulforhodamine B (Cat No. 018-10012: Wako Pure Chemical Industries, Ltd., Tokyo, Japan) solution in 1% acetic acid was added to each well, the plates were incubated for 30 min at room temperature. Unbound dye was removed by washing three times with distilled water and the plates were air dried. The bound stain was solubilized with 10 mM Tris base and the values of OD_515_ were measured by microtiter plate reader.

Percentages of net cell growth were calculated with the values of test growth in the presence of drug (Ti), growth control (C) and Tz according to the formula: [(Ti − Tz) ÷ (C − Tz)] × 100. Compound concentration required for 50% net growth inhibition (GI_50_) values were determined by non-linear curve fit analysis using the OriginPro (OriginLab Corporation, Northampton, MA, USA).

AlamaeBlue (Dainippon Pharmaceutical Co., Ltd., Tokyo, Japan) assay was used for suspension cell lines according to manufacturer’s protocol. In brief, one-tenth of AlamaeBlue was added into each well after 48 h incubation with the compound and the values of OD_570_ minus OD_600_ were measured by microtiter plate reader after 3 h incubation at 37 °C. Percentages of gross cell growth were calculated with the values of test growth in the presence of drug (Ti) and growth control (C) according to the formula: (Ti ÷ C) × 100.

#### Animal experiments

Animal experimental procedures in this study were in compliant with the regulations of Animal Welfare Committee in Novartis Institutes for BioMedical Research Tsukuba. The following mouse strains at the age of 5- to 6 weeks were purchased from Charles River (Yokohama, Japan): male BALB/c-nu/nu mice for MIA PaCa-2 subcutaneous inoculation; male C.B-17/IcrCrj-scid/scid mice for MIA PaCa-2 orthotopic inoculation; and female BALB/c mice for 4T1 orthotopic inoculation. Average body weight was 24 g with male BALB/c-nu/nu mice, and 18 g with male C.B-17/IcrCrj-scid/scid and female BALB/c mice. During inoculation, animals were anesthetized with isoflurane using a small animal anesthetizer. The mice bearing MIA PaCa-2 subcutaneous tumors and 4T1 orthotopic tumors with acceptable morphology, such as one round to oval shapes, excluding elongated thin rod-like shapes and split patterns, and size were randomized (7–8 mice per group). The mice bearing MIA PaCa-2 orthotopic tumors with acceptable luciferase activity detected by Xenogen system were randomized (8 mice per group). The mice were housed in a group of 4 to 5 per cage with access to water and food ad libitum in a normal light cycle room with specific pathogen free condition. At the end of the experiment, the mice were euthanized under terminal inhalation anesthesia with isoflurane followed by blood collection from abdominal vein.

#### Immunoblotting analysis

The tumor samples were pulverized in a CRYO-PRESS frozen cell crasher (#CP-100W, Microtec, Chiba, Japan). Samples were lysed by addition of 300 to 500 μL ice-cold T-PER Tissue Protein Extraction Reagent (#78510, Pierce, Rockford, IL, USA) containing protease inhibitors (#P2714, Sigma-Aldrich, St. Louis, MO, USA), and rotated at 4 °C for 30 min. Lysates were clarified by centrifugation at 15,000×*g* for 20 min at 4 °C. The antibodies used in this experiment are listed here: anti-phosphorylated ERK1/2, anti-ERK1/2, anti-phosphorylated Akt (pS473) and anti-Akt antibodies from Cell Signaling Technology (#9101, #9102, #9271 and #9272, respectively, Beverly, MA, USA); anti-phosphorylated FAK (pY397) antibody from Biosource International (#44-624, Camarillo, CA, USA); anti-FAK antibody from Upstate Biotechnology (#06-543, Lake Placid, NY, USA). Equal amounts of protein (tumor sample; 50 μg, cell lysates; 15 μg) were electrophoresed on 4–20% gradient SDS-PAGE gels (#301506, Dai-ichi pure chemicals, Tokyo, Japan) and transferred to an Immobilon-P PVDF membrane (#IPVH00010, Millipore, Bedford, MA, USA). Immunoblotting of transferred proteins was performed with appropriate antibodies (1:1000 dilutions) overnight at 4 °C. The membranes were washed in TBS-T (10 mM Tris–HCl, pH 7.4, 150 mM NaCl, 0.1% Tween 20) and incubated with HRP conjugated donkey anti-rabbit IgG with 1:1000 dilution (#NA9340V, Amersham, Arlington Heights, IL, USA) at room temperature for 1 h. The membranes were washed extensively, and the proteins were visualized by enhanced chemiluminescence (#34095, SuperSignal West Femto Maximum Sensitivity Substrate, Pierce, Rockford, IL, USA). Signals were quantified by utilizing a LAS-1000plus image analyzer and ImageGauge software (Fuji Film, Tokyo, Japan).

#### Compound treatment and statistical analyses

TAE226 was formulated in Sandimmune–Neoral Placebo drink solution and administered once daily through oral gavage in a volume of 10 mL/kg. Tumor volumes were calculated according to the formula: length × width^2^/2. In efficacy experiments, the treatments were initiated when the mean tumor volumes reached approximately 150 mm^3^ for MIA PaCa-2 subcutaneous tumors and approximately 70 mm^3^ for 4T1 tumors. 4T1 cells were inoculated subcutaneously into the mammary fat pad of female BALB/c mice. In case of MIA PaCa-2 orthotopic tumors, a stable line expressing luciferase was created and mice bearing tumors with acceptable luciferase activity detected by Xenogen system were randomized and used in the experiment. Treatment was started at 24 days (= day 0) after MIA PaCa-2 cells were surgically implanted into pancreas of male CB17-SCID mice. Tumors were weighed after normal pancreas tissues were removed at day 24. Since tumor weights at day 0 cannot be measured, T/C values are calculated only with the weights at the end of treatment. Note that a little normal pancreas tissues were still remained, although it was removed as much as possible. Statistical analyses were performed by SYSTAT (SYSTAT Software Inc., Point Richmond, CA, USA). Differences were considered to be significant when the probability value was < 0.05.

### Results and discussion

TAE226 was evaluated in a panel of 37 cancer cell lines comprising breast, prostate, lung, colon, stomach, pancreas, glioma, melanoma and myeloma to profile the in vitro anti-proliferative activity and showed a broad spectrum of activity in the panel with the mean GI_50_ value of 0.76 μmol/L, ranging from 0.14 to 3.6 μM (Table 1). TAE226 was effective in *P*-glycoprotein expressing MCF-7/ADR-RES with a comparable potency in the parent line MCF-7, suggesting that TAE226 is not a substrate of *P*-glycoprotein. Potent anti-proliferative activity of TAE226 against wide range of cancer cell lines including multi-drug resistant cells indicated that TAE226 is effective against tumors that are resistant to conventional anti-cancer drugs.

**Table 1:** Anti-proliferative activity of TAE226 against cancer cell lines

Cell origin	Cell line name	GI_50_ (μM)	n
Breast	MCF-7	1.2 ± 0.22	7
	MCF-7/ADR-RES	1.1 ± 0.23	4
	MDA-MB-231	0.56 ± 0.22	3
	MDA-MB-435	1.6 ± 0.044	3
	MDA-MB-453	1.5 ± 0.32	3
	4T1	0.16 ± 0.022	5
	MTF7	1.4 ± 0.26	3
Prostate	DU145	0.23 (0.20, 0.25)	2
	PC-3/M	0.83 ± 0.29	4
Lung	NCI-H23	0.29 (0.24, 0.35)	2
	NCI-H460	0.40 ± 0.028	4
	LLC	0.13 ± 0.0077	4
Colon	COLO205	0.21 (0.11, 0.30)	2
	HCT-15	0.71 ± 0.1	5
	HCT-116	0.42 ± 0.056	3
	SW620	0.51 ± 0.024	3
	WiDr	0.14 ± 0.030	5
Stomach	KATOIII	0.54 ± 0.064	3
Pancreas	BxPC-3	0.53 ± 0.11	3
	MIA PaCa-2	0.26 ± 0.062	4
	PANC-1	3.6 ± 0.93	3
	SUIT-2	0.18 ± 0.029	3
Glioma	A172	1.6 (2.2, 1.1)	2
	DBTRG-05MG	0.47 ± 0.054	3
	LN-18	0.96 ± 0.12	3
	LN-229	0.56 ± 0.043	3
	T98G	0.42 (0.21, 0.63)	2
	U-87 MG	1.0 (1.1, 0.97)	2
	U-118 MG	1.3 (0.69, 1.8)	2
	U-373 MG	1.4 (0.72, 2.0)	2
Melanoma	A375M	0.29 (0.15, 0.43)	2
	C32	1.8 ± 0.31	3
	C8161	0.90 (0.68, 1.1)	2
	SK-MEL-23	0.17 ± 0.0090	3
	SK-MEL-93	0.25 ± 0.050	4
	WM1158	0.33 ± 0.099	3
Myeloma	RPMI8226	0.33 ± 0.060	3

TAE226 concentrations causing 50% inhibition of net cell growth (GI_50_) are given. Results are expressed as mean ± SEM (except experiments with n = 2 where 2 individual values are included). Cell growth was assessed by sulforhodamine B staining for adhesive cell lines and by AlamarBlue for suspension cell lines. GI_50_ values were calculated after 48 h treatment with TAE226

**Fig. 1:**
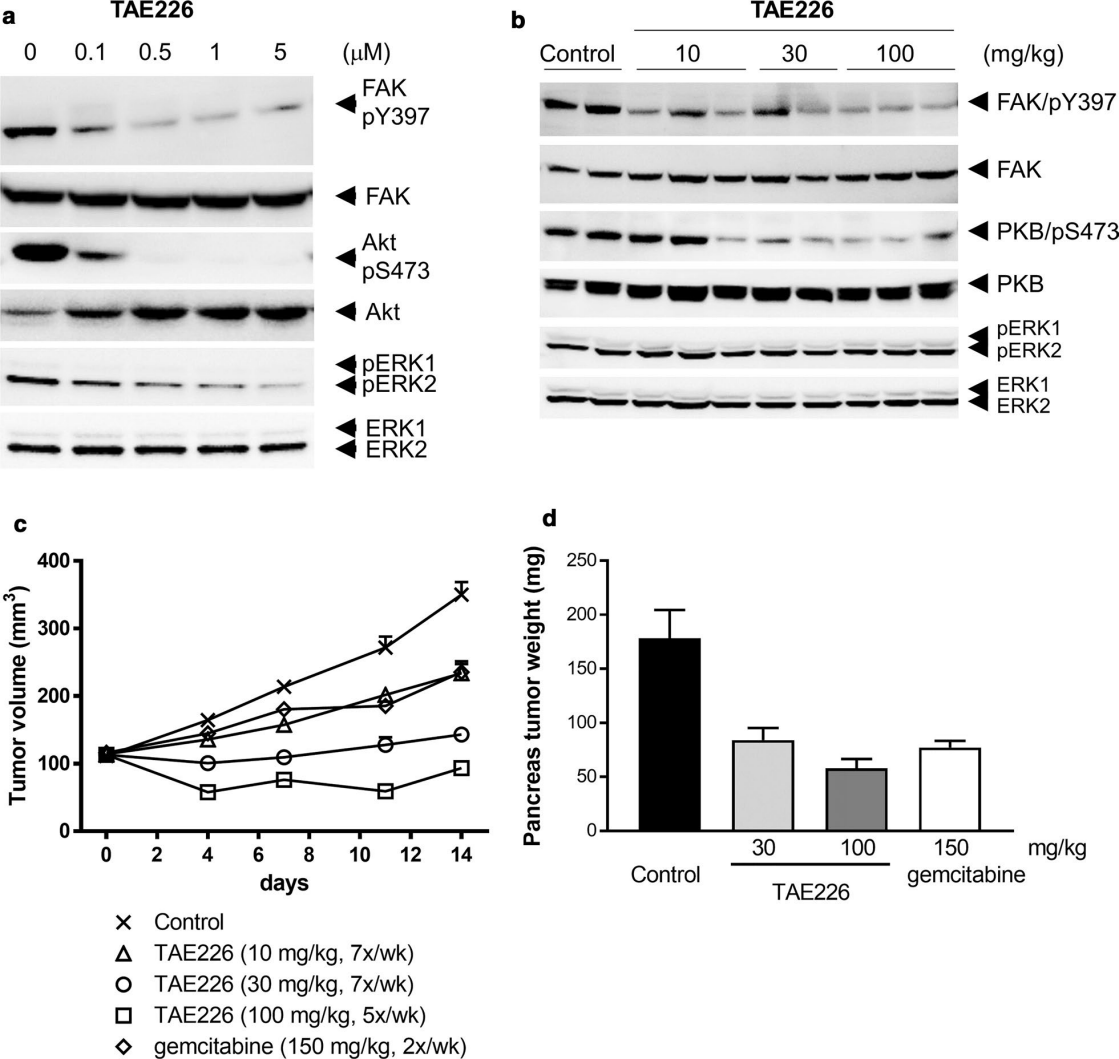
Effects of TAE226 on signaling pathways and tumor growth in MIA PaCa-2 models. **a** Effects of TAE226 on phosphorylation of FAK at Y397, Akt at S473 and ERK1/2 in MIA PaCa-2 cells treated for 1 h. **b** Effects of TAE226 on phosphorylation of FAK at Y397, Akt at S473 and ERK1/2 in MIA PaCa-2 tumors. Tumor samples were collected 3 h after administration of TAE226 at doses of 10, 30 and 100 mg/kg. Two or three animals were used for each group and each lane represents an individual animal. **c** Effects of TAE226 on MIA PaCa-2 subcutaneous tumor growth (n = 7). Tumor volume was calculated according to the formula: length × width^2^/2. **d** Effects of TAE226 on MIA PaCa-2 orthotopic tumor growth (n = 8). Values are expressed by mean ± SEM. Gemcitabine was administered intravenously twice weekly

Next, the effect of TAE226 on phosphorylation of FAK, Akt and ERK1/2 was evaluated in MIA PaCa-2 cells in vitro and tumors in vivo. Phosphorylation of FAK at Y397, Akt at S473 and ERK was observed in MIA PaCa-2 cells (Fig. 1a) and MIA PaCa-2 tumors (Fig. 1b). TAE226 inhibited phosphorylation of FAK at Y397, resulting in suppression of phosphorylation of Akt at S473 and ERK1/2 in MIA PaCa-2 cells 1 h after treatment (Fig. 1a). In MIA PaCa-2 tumors collected 3 h after administration of TAE226, TAE226 inhibited phosphorylation of FAK at Y397 and phosphorylation of Akt at S473 at all doses tested, but the effect on phosphorylation of ERK1/2 was not clear in vivo (Fig. 1b).

Anti-tumor activity of TAE226 was evaluated in the MIA PaCa-2 subcutaneous and orthotopic xenograft models. Oral administration of TAE226 efficiently inhibited MIA PaCa-2 tumor growth at all doses tested. After 14 days treatment, T/C values were 50% at 10 mg/kg and 13% at 30 mg/kg, qd for 7×/week (Fig. 1c, Additional file 2: Table S2). At a dose of 100 mg/kg, qd for 5×/week, tumor regression (17%) was observed (Fig. 1c). A reference anti-cancer agent for pancreas carcinoma, gemcitabine at the maximum tolerated dose was moderately effective against MIA PaCa-2 model (T/C: 50%), which was similar to the report [26]. TAE226 also inhibited MIA PaCa-2 orthotopic tumor growth in pancreas dose-dependently (Fig. 1d). Body weight loss was not observed in TAE226-treated group in both experiments. Efficacy of TAE226 at 10 mg/kg in the subcutaneous tumor and at 30 mg/kg in the orthotopic tumor was comparable to that of gemcitabine at the maximum tolerated dose, and therefore TAE226 showed superior safety margin to gemcitabine.

**Fig. 2:**
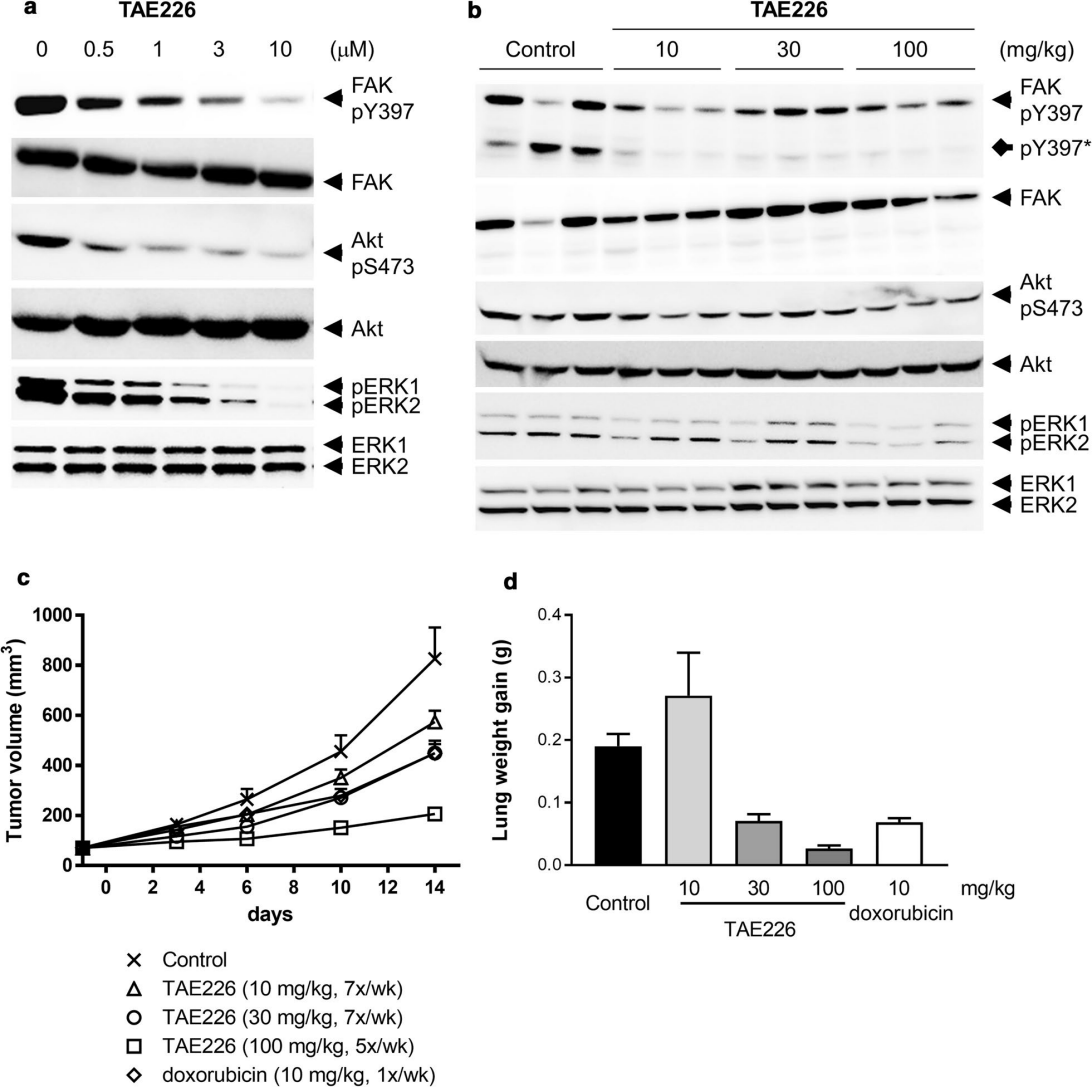
Effects of TAE226 on signaling pathways and tumor growth in 4T1 models. **a** Effects of TAE226 on phosphorylation of FAK at Y397, Akt at S473 and ERK1/2 in 4T1 cells treated for 1 h. **b** Effects of TAE226 on phosphorylation of FAK at Y397, Akt at S473 and ERK1/2 in 4T1 tumors. Tumor samples were collected 3 h after administration of TAE226 at doses of 10, 30 and 100 mg/kg. Two or three animals were used for each group and each lane represents an individual animal. Asterisk indicates degraded phospho-FAK in 4T1 tumor probably due to insufficient inhibition of non-specific protease inhibition. **c** Effects of TAE226 on 4T1 tumor growth (n = 8). Tumor volume was calculated according to the formula: length × width^2^/2. **d** Effects of TAE226 on metastasis. Values are expressed by mean ± SEM. Doxorubicin was administered intravenously once weekly. Values are expressed by mean ± SEM

Then, the effect of TAE226 on phosphorylation of FAK, Akt and ERK1/2 was evaluated 4T1 cells in vitro and tumors in vivo. Phosphorylation of FAK at Y397, Akt at S473 and ERK was observed in 4T1cells (Fig. 2a) and 4T1 tumors (Fig. 2b). TAE226 inhibited phosphorylation of FAK at Y397, resulting in suppression of phosphorylation of Akt at S473 and ERK1/2 in 4T1 cells 1 h after treatment (Fig. 2a). In 4T1 tumors collected 3 h after administration of TAE226, TAE226 inhibited phosphorylation of FAK at Y397 and phosphorylation of Akt at S473 at all doses tested, but the effect on phosphorylation of ERK1/2 was not clear in vivo (Fig. 2b). Degradation products of FAK, which were also detected by anti-N-terminal FAK antibody (data not shown), were observed in the 4T1 experiment (indicated by diamond arrow: pY397*).

4T1 is a highly malignant murine carcinoma cell line and spontaneously metastasizes to the lung, liver, lymph nodes and brain while the primary tumor is growing in situ [5]. The tumor growth and metastatic spread of 4T1 cells in BALB/c mice very closely resembles the situation in breast carcinoma patients. In the model, anti-tumor and anti-metastasis activities can be assessed at the same time. Oral administration of TAE226 inhibited 4T1 tumor growth and metastasis to the lung in a dose-dependent manner. Primary tumor growth was significantly inhibited by the dose of as low as 10 mg/kg. After 14 days treatment, T/C values were 67%, 50% and 18% at 10 mg/kg, 30 mg/kg, qd for 7×/week and 100 mg/kg, qd for 5×/week, respectively (Fig. 2c, Additional file 3: Table S3). Metastasis to lung was prevented with T/C values of 37% and 14% by the 30 mg/kg dose qd×7 and 100 mg/kg qd×5, respectively (Fig. 2d, Additional file 3: Table S3). The compound was well tolerated in mice as determined by measuring changes in body weight. A reference anti-cancer agent for breast carcinoma, doxorubicin, was moderately effective against 4T1 model (T/C: 50% primary tumor, 36% metastasis) at the maximum tolerated dose (Fig. 2c, d, Additional file 3: Table S3). Efficacy of TAE226 at 30 mg/kg was comparable to that of doxorubicin at the maximum tolerated dose, and therefore TAE226 showed superior safety margin to doxorubicin.

As inhibition of tumor growth by TAE226 appears to correlate with inhibition of FAK and Akt as a surrogate of IGF-1R signaling pathway, TAE226 represents a novel class of selective and small molecule kinase inhibitor with a potent in vivo activity.

## Limitations

Further characterization of TAE226 will be required, for example, in pharmacokinetics and safety assessment to define safety margin as well as in patient-derived cells and tumors to stratify target patient population and tumor types. Some methods used in this study may be outdated since the profiling was performed in early 2000.

## Additional files

**Additional file 1: Table S1.** Plating density of cell lines used in panel screening.

**Additional file 2: Table S2.** Effects of TAE226 on MIA PaCa-2 subcutaneous tumor growth and body weight change.

**Additional file 3: Table S3.** E﻿ffects of TAE226 on 4T1 primary tumor growth, body weight change and lung metastasis.

## Data Availability

The datasets used in the current study are available from the corresponding author by request.

## Abbreviations

BWC: body weight change
ERK: extracellular signal-regulated kinase
FAK: focal adhesion kinase
GI_50_: growth inhibition of 50%
IGF-1R: insulin-like growth factor 1 receptor
LWG: lung weight gain
T/C: tumor/control
TV: tumor volume
