# Supplementary material for: Pharmacological profiling of a dual FAK/IGF-1R kinase inhibitor TAE226 in cellular and in vivo tumor models
Source: BMC Res Notes. 2019 Jun 18;12:347. doi: 10.1186/s13104-019-4389-7 (PMC6582604; doi:10.1186/s13104-019-4389-7)
Supplement: Supplementary file 1 — Additional file 1: Table S1. Plating density of cell lines used in panel screening. [file 13104_2019_4389_MOESM1_ESM.docx]

**Table S1: Plating density of cell lines used in panel screening**

| Cell line name | Cells/well | Origin |
| --- | --- | --- |
| MCF-7 | 10,000 | MD Anderson Cancer Center |
| MCF-7/ADR-RES | 15,000 | MD Anderson Cancer Center |
| MDA-MB-231 | 20,000 | MD Anderson Cancer Center |
| MDA-MB-435 | 15,000 | MD Anderson Cancer Center |
| MDA-MB-453 | 10,000 | MD Anderson Cancer Center |
| 4T1 | 5,000 | Osaka Univ. |
| MTF7 | 10,000 | MD Anderson Cancer Center |
| DU145 | 10,000 | MD Anderson Cancer Center |
| PC-3/M | 7,500 | MD Anderson Cancer Center |
| NCI-H23 | 20,000 | ATCC |
| NCI-H460 | 5,000 | ATCC |
| LLC | 5,000 | MD Anderson Cancer Center |
| COLO205 | 10,000 | Dainippon Pharmaceutical |
| HCT-15 | 10,000 | Dainippon Pharmaceutical |
| HCT-116 | 5,000 | ECACC |
| SW620 | 10,000 | Dainippon Pharmaceutical |
| WiDr | 10,000 | Dainippon Pharmaceutical |
| KATOIII | 10,000 | MD Anderson Cancer Center |
| BxPC-3 | 10,000 | ATCC |
| MIA PaCa-2 | 10,000 | ECACC |
| PANC-1 | 5,000 | Dainippon Pharmaceutical |
| SUIT-2 | 10,000 | Kyushu Univ. |
| A172 | 15,000 | ECACC |
| DBTRG-05MG | 10,000 | ATCC |
| LN-18 | 10,000 | ATCC |
| LN-229 | 10,000 | ATCC |
| T98G | 15,000 | ECACC |
| U-87 MG | 15,000 | ATCC |
| U-118 MG | 10,000 | ATCC |
| U-373 MG | 15,000 | ECACC |
| A375M | 5,000 | MD Anderson Cancer Center |
| C32 | 10,000 | ATCC |
| C8161 | 15,000 | MD Anderson Cancer Center |
| SK-MEL-23 | 10,000 | MD Anderson Cancer Center |
| SK-MEL-93 | 10,000 | MD Anderson Cancer Center |
| WM1158 | 10,000 | MD Anderson Cancer Center |
| RPMI8226 | 20,000 | ATCC |
