# Supplementary material for: Pharmacological profiling of a dual FAK/IGF-1R kinase inhibitor TAE226 in cellular and in vivo tumor models
Source: BMC Res Notes. 2019 Jun 18;12:347. doi: 10.1186/s13104-019-4389-7 (PMC6582604; doi:10.1186/s13104-019-4389-7)
Supplement: Supplementary file 2 — Additional file 2: Table S2. Effects of TAE226 on MIA PaCa-2 subcutaneous tumor growth and body weight change. [file 13104_2019_4389_MOESM2_ESM.docx]

**Table S2: Effects of TAE226 on MIA PaCa-2 subcutaneous tumor growth and body weight change**

| Group | Regimen | Dose  (mg/kg) | Delta TV  (mm^3^) | Delta T/C  (%) | Regression  (%) | BWC (%) | Alive/  Total |
| --- | --- | --- | --- | --- | --- | --- | --- |
| Control | 7×/wk | - | 239 ± 21 | - | - | 10.3 ± 0.9 | 7/7 |
| TAE226 | 7×/wk | 10 | 120 ± 11 | 50^**^ | - | 9.9 ± 0.3 | 7/7 |
|  | 7×/wk | 30 | 30 ± 7 | 13^**^ | - | 6.2 ± 0.9^*^ | 7/7 |
|  | 5×/wk | 100 | -19 ± 1 | -8^**^ | 17^##^ | 7.0 ± 1.2^*^ | 7/7 |
| gemcitabine | 2×/wk | 150 | 119 ± 13 | 5 ^**^ | - | 5.8 ± 1.1^*^ | 7/7 |

Treatment was started when tumor volumes had reached approximately 150 mm^3^. Final tumor volumes were recorded after 14 days treatment. Delta T/C (%) was calculated according to the formula: (mean change tumor volumes of treated animals / mean changes of tumor volumes of control animals) × 100. Regressions (%) were calculated according to the formula: (mean changes of tumor volume / mean tumor volume at start of treatment) × 100. Values of Delta TV (tumor volume) and BWC (body weight change) are expressed by means ± SEM. ^*^: *P* < 0.05, ^**^: *P* < 0.01 versus Control (Dunnett’s test following ANOVA); ^##^: *P* < 0.01 versus each tumor volume at day0 (Paired t-test).
