# Supplementary material for: Pharmacological profiling of a dual FAK/IGF-1R kinase inhibitor TAE226 in cellular and in vivo tumor models
Source: BMC Res Notes. 2019 Jun 18;12:347. doi: 10.1186/s13104-019-4389-7 (PMC6582604; doi:10.1186/s13104-019-4389-7)
Supplement: Supplementary file 3 — Additional file 3: Table S3. E﻿ffects of TAE226 on 4T1 primary tumor growth, body weight change and lung metastasis. [file 13104_2019_4389_MOESM3_ESM.docx]

**Table S3: Effects of TAE226 on 4T1 primary tumor growth, body weight change and lung metastasis**

| Group | Regimen | Dose  (mg/kg) | at day14 | | | | at day21 | | |
| --- | --- | --- | --- | --- | --- | --- | --- | --- | --- |
|  |  |  | Delta TV  (mm^3^) | Delta  T/C  (%) | BWC  (%) | Alive/  Total | LWG  (mg) | Delta  T/C  (%) | Alive/  Total |
| Control | 7×/wk | - | 775 ± 121 | - | 6.4 ± 1.0 | 8/8 | 190 ± 20 | - | 8/8 |
| TAE226 | 7×/wk | 10 | 504 ± 41 | 67^*^ | 4.7 ± 1.3 | 8/8 | 271 ± 69 | 142 | 6/8 |
|  | 7×/wk | 30 | 380 ± 35 | 50^**^ | 4.2 ± 1.6 | 8/8 | 71 ± 11 | 37^*^ | 8/8 |
|  | 5×/wk | 100 | 136 ± 22 | 18^**^ | 0.8 ± 1.6^*^ | 8/8 | 27 ± 5 | 14^**^ | 8/8 |
| doxorubicin | 1×/wk | 10 | 377 ± 46 | 50^#^ | -10.4 ± 0.9^##^ | 8/8 | 68 ± 7 | 36^##^ | 8/8 |

Treatment was started when tumor volumes had reached approximately 70 mm^3^. Tumor volumes for statistical analysis were recorded after 14 days treatment because acceleration of necrosis in the central part of tumor is obvious after day14. Delta T/C (%) was calculated according to the formula: (mean change tumor volumes of treated animals / mean changes of tumor volumes of control animals) x 100. Values of Delta TV (tumor volume), BWC (body weight change) and LWG (lung weight gain) are expressed by Mean ± SEM. *: *P* < 0.05, ^**^: *P* < 0.01 versus Control (Dunnett’s test following ANOVA); ^#^: *P* < 0.05, ^##^: *P* < 0.01 versus Control (Student’s t-test).
